# Bacterial Growth of Uropathogenic Escherichia coli in Pooled Urine Is Much Higher than Predicted from the Average Growth in Individual Urine Samples

**DOI:** 10.1128/spectrum.02016-22

**Published:** 2022-09-26

**Authors:** Jacob Hogins, Ethan Fan, Zheyar Seyan, Sam Kusin, Alana L. Christie, Philippe E. Zimmern, Larry Reitzer

**Affiliations:** a Department of Biological Sciences, University of Texas at Dallas, Richardson, Texas, USA; b Department of Urology, University of Texas Southwestern Medical School, Dallas, Texas, USA; University of Texas Southwestern Medical Center

**Keywords:** *Escherichia coli*, bacterial growth, urinary tract infection

## Abstract

Urinary tract infections (UTIs), mostly caused by uropathogenic E. coli (UPEC), affect most women, and often recur. Genomic and transcriptomic analyses have not identified a common set of virulence genes, which has suggested complex host-pathogen interactions and multiple virulence mechanisms. One aspect of the host-pathogen interaction is rapid UPEC growth in urine *in vivo*. When bacterial growth in urine is studied *in vitro*, urine is pooled, which is assumed to diminish individual variation. We grew one nonpathogenic and two pathogenic E. coli strains in urine from individuals who never had a UTI, had a UTI history but no current infection, and had a UTI history with a current infection. Bacterial growth showed large variations in individual urine samples, and pooled urine often supported significantly more growth than the average growth from individual urine samples. Total nutrient content tended to be higher in current group urine samples than the never and history grouped samples urine. We propose that pooling optimizes a nutrient mixture in the never and history group urine samples, which are often studied, whereas urine from current group individuals may have a more optimal nutrient mixture because of additional nutrient sources. We conclude that a pooled urine is not “an average urine sample,” and that the best comparisons of results between labs using pooled urine would also include results with a standardized synthetic urine.

**IMPORTANCE** Urinary tract infections (UTIs) will affect most women, can recur especially in postmenopausal women, and can become antibiotic recalcitrant. Escherichia coli causes most community-acquired UTIs and recurrent UTIs. Current theories of virulence, based on studies of UTI-associated E. coli, propose multiple virulence mechanisms and complex host-pathogen interactions. Studies of bacterial growth in urine samples—one aspect of the host-pathogen interaction—invariably involve pooled urine that are assumed to eliminate variations between individuals. Our results show that a pooled urine is not necessarily an average urine sample, and we suggest that quantitative and qualitative variations in nutrient content are the basis for this discrepancy. Knowledge of growth-promoting urinary components is important for understanding host-pathogen interactions during UTIs and could contribute to developing nonantibiotic-based therapies.

## OBSERVATION

**FIG 1:**
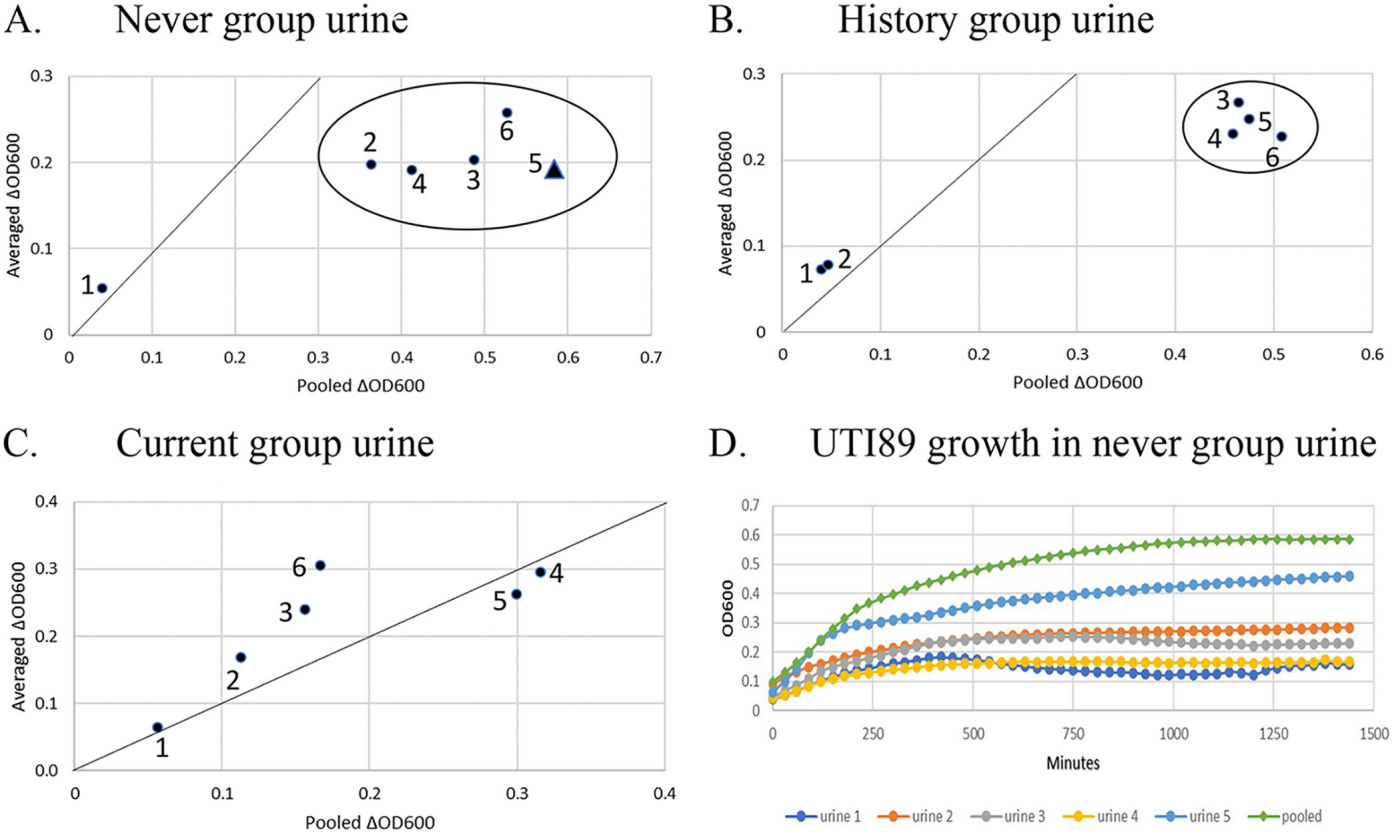
Comparative growth in pooled urine versus the average of individuals samples. (A) Growth in the never group urine; (B) growth in the history group urine; and (C) growth in the current group urine. (D) Growth of UTI89 in the never group urine with a high inoculation density. The numbered labels refer to the strains tested and initial inoculation density: 1, W3110 low; 2, W3110 high; 3, UTI89 low; 4 UTI89 high; 5, LRPF007 low; and 6, LRPF007 high. For the circled symbols, growth in pooled urine was significantly different (*P* < 0.05) from the average growth of the individual samples. The triangle in section A is from the growth curves shown in section D. Each group had five samples, and all growth cultures were performed in triplicate. The average growth in the individual urine samples was from the average of the 5 triplicate averages. For statistical comparisons, the three separate cultures for the pooled urine were compared to the 5 triplicate averages for the individual urine cultures. F-tests were used to analyze if variance was unequal between the individual versus pooled urine. If the F-test was significant, the heteroscedastic *t* test was used to test for differences in the means; if the F-test was not significant, the homoscedastic *t* test was used. The diagonal line is expected if the ratio is one, i.e., growth in pooled urine is the same as the average of the individual urine samples.

UTI progression involves several processes, including rapid bacterial growth in urine (1). The failure of recent genomic and transcriptomic analyses to identify a common set of virulence factor genes in UPEC led Hultgren and colleagues to propose that UPEC strains have multiple virulence mechanisms that are based on a complex interplay between bacterial and host properties (2). Urinary composition is one component of the host-pathogen interaction which *prima facie* is a vital component. Recent studies that involve growth in urine have used pooled urine from 2 to 10 healthy individuals (average 5.6) (3–10). Pooling is assumed to eliminate variations between individuals which implies limited variation. This assumption predicts that growth in a pooled urine will be similar to the average growth in individual urine samples. To evaluate this prediction, we compared growth in pooled urine to the average growth from individual samples. Urine samples were collected from five postmenopausal women in “never” (never had a UTI), “history” (have a history of UTIs but asymptomatic and negative urinalysis), and “current” (have a history of UTIs and an active symptomatic UTI) groups. We grew three bacterial strains in each urine sample: W3110, a nonpathogenic lab strain; UTI89, a model UPEC strain (11); and LRPF007, a recently isolated UPEC strain from a patient with recurrent UTIs. We analyzed the ΔOD600 as a measure of growth instead of doubling time because few cultures had a constant exponential growth rate for more than one generation even with 30-minute sampling intervals. The ratio of growth in pooled urine versus the average growth in individual urine samples often deviated from 1.0 (the diagonal lines in Fig. 1A to C). The greatest deviations were from growth of the two UPEC strains in never and history group urine samples, and the differences were statistically significant (*P* < 0.05). UPEC growth in pooled urine could be greater than growth in any single urine sample (Fig. 1D).

**FIG 2:**
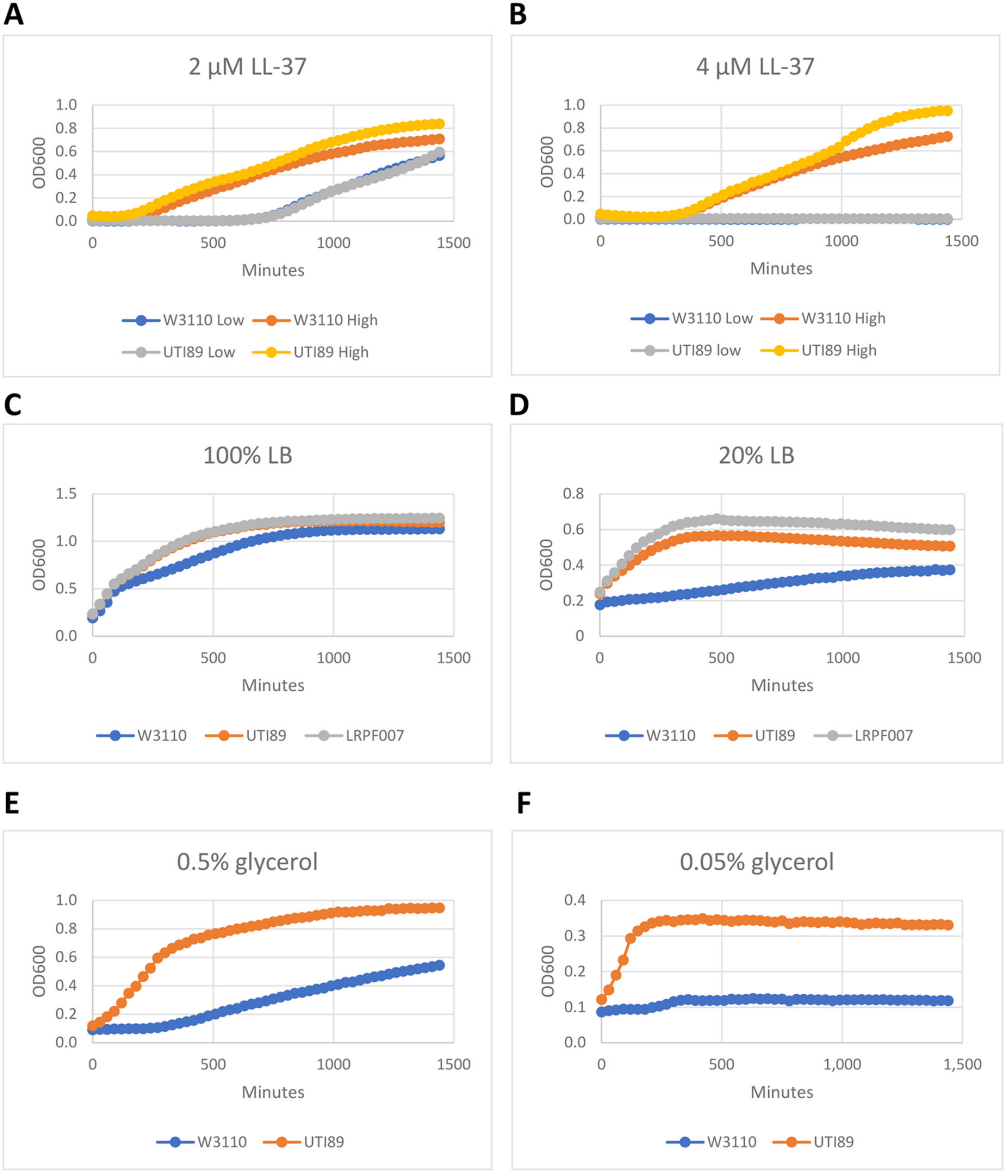
Effect of the antimicrobial peptide LL-37 and media dilution on UTI89, LRPF007, and W3110. Growth with low and high inoculation densities with 2 μM LL-37 (A) and 4 μM LL-37 (B). Growth in 100% LB (C) and 20% LB (D). Growth in a minimal medium with 0.5% glycerol as sole carbon source (E) and 0.05% glycerol as sole carbon source (F).

To understand the basis for the growth synergism, we examined the two general types of factors that affect bacterial growth in urine: inhibitory factors and nutrient content. A characteristic feature of growth inhibitory factors (IFs), such as antimicrobial peptides and antibiotics, is the so-called inoculation density effect, i.e., the initial inoculation density determines the final cell density (12). A pronounced inoculation effect is apparent for W3110 and UTI89 with the antimicrobial peptide LL-37 at 2 and 4 μM (Fig. 2A and B) but is less apparent at ≤1.3 μM (not shown). Because of this effect, we analyzed growth in all urine samples after a low- and high-density inoculation as an indirect test for urinary IFs. We define low- and high-density inoculations as initial OD600s of 0.004 and 0.02, respectively, and the final growth ratio after a high versus low inoculation density as the HL ratio. For W3110, the HL ratios were high and variable which suggests sensitivity to IFs and substantial IF variation between individual urine samples (Table S1). For the UPEC strains, the average HL ratios were near 1.0 regardless of patient group (Table S1), which implies resistance to the IFs in the urine samples and that UPEC growth is a measure of utilizable nutrient content. The comparative growths of the two UPEC strains in current, never, and history group urine samples had ratios of approximately 3 to 2 to 1 (Table S2). For the current versus history groups, which has the highest ratio, the results achieve statistical significance for the high-density inoculations but not the low-density inoculations. In the nutrient-poor history group urine samples, both UPEC strains grew at least 2-fold better than W3110 (not shown). The UPEC strains also grew better than W3110 in diluted minimal and rich media (Fig. 2D to F), which shows that the UPEC strains utilize low levels of nutrients better than W3110.

### Urine collection and processing.

Following IRB approval from both University of Texas Southwestern Medical School and University of Texas at Dallas, midstream urine samples were collected from postmenopausal women who had not been on antibiotics for at least 4 weeks. The urine was frozen at −80C. For the growth experiments, urine was thawed, centrifuged to remove particulates, and sterilized with a 0.2 μm filter. Each well in a microtiter dish received 0.2 mL urine.

### Bacterial strains.

W3110 is a commonly used nonpathogenic lab strain. UTI89 is a model UPEC strain (11). LRPF007 is a UPEC strain from a patient with RUTIs which was isolated after plating on ChromeAgar, streaked on l-broth to obtain single colonies, and cells from a single colony were grown in l-broth. Glycerol was added to the culture which was frozen at -80 C. The strain was determined to be E. coli and phylotyped as described (13).

### Bacterial growth.

Bacterial strains were streaked on an l-broth agar plate and grown overnight. A single colony was inoculated into 1 mL l-broth for 2 h in a 15 mL glass tubes at 250 rpm, centrifuged and resuspended in PBS three times to remove residual medium, resuspended in PBS, and then diluted into urine samples. Bacteria were grown in triplicate wells and optical density at 600 nm was measured in BioTek plate readers. The ΔOD600s ranged from 0.023 to 0.424. For cultures with a ΔOD600 > 0.1, the initial doubling time varied from 51 to 207 min and did not correlate with the ΔOD600. For the ΔOD600, the average coefficient of variation (the ratio of the standard deviation to the average of the triplicate determinations) was 13%.

## References

[1] Flores-Mireles AL, Walker JN, Caparon M, Hultgren SJ. 2015. Urinary tract infections: epidemiology, mechanisms of infection and treatment options. Nat Rev Microbiol 13:269–284. doi:10.1038/nrmicro3432. 25853778 PMC4457377

[2] Schreiber H, Conover MS, Chou WC, Hibbing ME, Manson AL, Dodson KW, Hannan TJ, Roberts PL, Stapleton AE, Hooton TM, Livny J, Earl AM, Hultgren SJ. 2017. Bacterial virulence phenotypes of *Escherichia coli* and host susceptibility determine risk for urinary tract infections. Sci Transl Med 9. doi:10.1126/scitranslmed.aaf1283. PMC5653229 28330863

[3] Snyder JA, Haugen BJ, Buckles EL, Lockatell CV, Johnson DE, Donnenberg MS, Welch RA, Mobley HL. 2004. Transcriptome of uropathogenic *Escherichia coli* during urinary tract infection. Infect Immun 72:6373–6381. doi:10.1128/IAI.72.11.6373-6381.2004. 15501767 PMC523057

[4] Roos V, Ulett GC, Schembri MA, Klemm P. 2006. The asymptomatic bacteriuria *Escherichia coli* strain 83972 outcompetes uropathogenic *E. coli* strains in human urine. Infect Immun 74:615–624. doi:10.1128/IAI.74.1.615-624.2006. 16369018 PMC1346649

[5] Alteri CJ, Mobley HL. 2007. Quantitative profile of the uropathogenic *Escherichia coli* outer membrane proteome during growth in human urine. Infect Immun 75:2679–2688. doi:10.1128/IAI.00076-07. 17513849 PMC1932884

[6] Alteri CJ, Smith SN, Mobley HL. 2009. Fitness of *Escherichia coli* during urinary tract infection requires gluconeogenesis and the TCA cycle. PLoS Pathog 5:e1000448. doi:10.1371/journal.ppat.1000448. 19478872 PMC2680622

[7] Hagan EC, Lloyd AL, Rasko DA, Faerber GJ, Mobley HL. 2010. Escherichia coli global gene expression in urine from women with urinary tract infection. PLoS Pathog 6:e1001187. doi:10.1371/journal.ppat.1001187. 21085611 PMC2978726

[8] Hancock V, Vejborg RM, Klemm P. 2010. Functional genomics of probiotic *Escherichia coli* Nissle 1917 and 83972, and UPEC strain CFT073: comparison of transcriptomes, growth and biofilm formation. Mol Genet Genomics 284:437–454. doi:10.1007/s00438-010-0578-8. 20886356

[9] Vejborg RM, de Evgrafov MR, Phan MD, Totsika M, Schembri MA, Hancock V. 2012. Identification of genes important for growth of asymptomatic bacteriuria *Escherichia coli* in urine. Infect Immun 80:3179–3188. doi:10.1128/IAI.00473-12. 22753377 PMC3418751

[10] Greene SE, Hibbing ME, Janetka J, Chen SL, Hultgren SJ. 2015. Human urine decreases function and expression of type 1 pili in uropathogenic *Escherichia coli*. mBio 6:e00820. doi:10.1128/mBio.00820-15. 26126855 PMC4488945

[11] Mulvey MA, Schilling JD, Hultgren SJ. 2001. Establishment of a persistent *Escherichia coli* reservoir during the acute phase of a bladder infection. Infect Immun 69:4572–4579. doi:10.1128/IAI.69.7.4572-4579.2001. 11402001 PMC98534

[12] Loffredo MR, Savini F, Bobone S, Casciaro B, Franzyk H, Mangoni ML, Stella L. 2021. Inoculum effect of antimicrobial peptides. Proc Natl Acad Sci USA 118. doi:10.1073/pnas.2014364118. PMC8166072 34021080

[13] Clermont O, Bonacorsi S, Bingen E. 2000. Rapid and simple determination of the Escherichia coli phylogenetic group. Appl Environ Microbiol 66:4555–4558. doi:10.1128/AEM.66.10.4555-4558.2000. 11010916 PMC92342

