# Supplementary material for: Bacterial Growth of Uropathogenic Escherichia coli in Pooled Urine Is Much Higher than Predicted from the Average Growth in Individual Urine Samples
Source: Microbiol Spectr. 2022 Sep 26;10(5):e02016-22. doi: 10.1128/spectrum.02016-22 (PMC9603375; doi:10.1128/spectrum.02016-22)
Supplement: Supplemental file 1 — Tables S1 and S2. Download spectrum.02016-22-s0001.pdf, PDF file, 0.1 MB [file spectrum.02016-22-s0001.pdf]

SUPPLEMENTAL TABLES

Supplemental Table 1. The average HL ratio for each patient group. The indicated ratio is derived from the average HL ratio of 5 current and never group samples and 9 history group samples.

	W3110	UTI89	LRPF007
Current	3.9 ± 4.0	1.2 ± 0.5	1.2 ± 0.3
History	2.8 ± 3.8	1.3 ± 1.0	1.0 ± 0.4
Never	4.3 ± 2.5	1.0 ± 0.1	1.6 ± 0.7

Supplemental Table 2. Comparative growth in urine from different patient groups. The indicated ratios are derived from 5 current and never group samples and 9 history group samples. Because we are assessing nutrient content, the ΔOD600s were normalized to creatinine to adjust for hydration. F-tests indicated that the variances were not statistically different between the individual versus pooled urine samples, and the homoscedastic *t*-test was used.

	UTI89		LRPF007		Average ratio
Inoculation	Low	High	Low	High	
Current vs History	2.5 (0.090)	3.0 (0.025)	3.0 (0.15)	2.9 (0.042)	2.85
Current vs Never	1.4 (0.49)	1.6 (0.35)	1.5 (0.39)	1.5 (0.73)	1.5
Never vs History	1.7 (0.33)	1.9 (0.19)	1.8 (0.31)	2.4 (0.14)	1.95
